# Preparation and Antitumor Activity of CS5931, A Novel Polypeptide from Sea Squirt *Ciona Savignyi*

**DOI:** 10.3390/md14030047

**Published:** 2016-03-21

**Authors:** Xiaoshuang Chen, Huanli Xu, Bo Li, Feng Wang, Xiaoliang Chen, Dexin Kong, Xiukun Lin

**Affiliations:** 1 chenxiaoshuang2012@126.comkongdexin@tijmu.edu.cn; 2 xhl52@msn.comchen911_1@163.com; 3 libo991@126.comwangfeng9911@126.com

**Keywords:** CS5931, protein expression, renaturation, antitumor activity

## Abstract

CS5931 is a novel anticancer agent isolated from the sea squirt *Ciona savignyi*. However, its content in the species is very low, and developing a novel approach for production of the polypeptide is promising. In the present study, we expressed and purified the polypeptide from *E. coli*, and the fermentation conditions were studied using response surface methodology. The yield of CS5931 was increased from 2.0 to 7.5 mg/L. The denaturing and renaturation conditions were also studied. Using the optimized renaturation condition, the anticancer activity of refolding CS5931 was increased significantly; the value of IC_50_ was decreased from 23.2 to 11.6 μM. *In vivo* study using xenograft nude mice bearing HCT116 cancer cells revealed that CS5931 was able to inhibit the growth of tumor significantly. The study provides a useful approach for obtaining enough amount of CS5931 for further study. This study is also important for developing the polypeptide as a novel anticancer agent.

## 1. Introduction

Peptides play a crucial role in many physiological processes [1]. However, a small number of peptides have been commercialized as drugs because of their limited low yield and poor bioavailability. Recombinant expression of peptides has become the most promising family of compounds with potential application for human diagnosis and therapy. In recent years, a lot of peptides with potent antitumor activity have been isolated from marine organisms, and some of them have been successfully used clinically [2,3]. Aplidine (dehydrodidemnin B, DDB, Aplidin), a cyclic depsipeptide is isolated from the Mediterranean tunicate *Aplidium albicans*. Breast, melanoma and non-small-cell lung cancer appear to be sensitive to low concentrations of Aplidine [4,5]. Dolastatin 10, a linear pentapeptide containing several unique amino acid subunits is derived from the marine mollusk *Dolabella auricularia*; it is the most potent member of a large class of related peptides [6,7]. Dolastatin 10 causes formation of a cold-stable tubulin aggregate at higher drug concentrations. Dolastatin 10 strongly inhibits microtubule assembly, tubulin-dependent GTP hydrolysis, and the binding of vinca alkaloids to tubulin. A conjugate [8], called Brentuximab vedotin (SGN-35, Adcetris) is designed, which consists of the anti-CD30 monoclonal antibody SGN-35 has been approved by the US Food and Drug Administration (FDA) in 2011 for the treatment of relapsed or refractory Hodgkin’s lymphoma (HL) and anaplastic large cell lymphoma (ALCL) [9,10].

In our previous study, we isolated a novel antitumor polypeptide termed CS5931 from the sea squirt *Ciona savignyi* [11]; it shares high homology with *Ciona intestinalis* GRN and is conserved during evolution [12]. The polypeptide shows specific inhibition effect on the growth of several tumor cells *in vitro*, especially on human colon carcinoma HCT116 cells. CS5931 induces cell death via mitochondrial pathway as revealed by Hoechst 33258 staining experiment as well as flow cytometric analysis [12,13]. However, due to its low content in the species, developing an effective approach for preparing the polypeptide is promising. On the other hand, prediction of 3D structure of the polypeptide reveals that CS5931 consists of six disulfide bonds and two beta-hairpins, similar as human GRN-A [12]. Therefore, constructing suitable conditions for the refolding of the polypeptide is also important for keeping the anticancer activity of CS5931.

In the present study, we developed an effective approach for expressing the polypeptide in *E. coli*, and the conditions for fermentation of CS5931 were studied by central composite design (CCD). The denaturing and renaturing conditions of CS5931 were also optimized. *In vivo* study reveals that the recombinant polypeptide possesses potent antitumor activity against xenograft nude mice bearing human HCT116 carcinoma.

## 2. Results

### 2.1. Expression and Purification of the His-Tag CS5931

**Figure 1:**
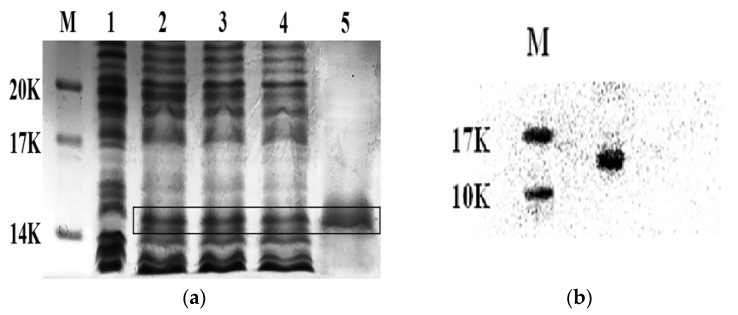
(**a**) SDS-PAGE analysis of recombinant CS5931 expression in BL21 (DE3) cells. The plasmid pET28a/CS5931 was transformed into *E. coli* BL21 (DE3). After being cultured at 37 °C for 16 h, the whole cell extract was resolved using SDS-PAGE in the absence (lane 1) and presence of IPTG induction (lane 2, 3). Lane 4 represented the total protein in the supernatant and lane 5 indicated the purified protein with the AKTA; (**b**) Western blot analysis of the purified polypeptide.

The plasmid pET28a/CS5931, synthesized in our laboratory previously [12] was transformed into *E. coli* BL21 (DE3). After isopropylthio-beta-galactoside (IPTG) induction overnight, the expression of CS5931 was determined using SDS-PAGE. The results showed that CS5931 was mostly expressed in the soluble fraction (Figure 1a, lane 2–4). The polypeptide was purified using Ni^2+^-NTA agarose columns. SDS-PAGE analysis showed that the CS5931 fusion protein displayed a clear band with an apparent molecular weight of 15 kDa (Figure 1a, lane 5). Of note, the theoretical molecular weight of CS5931 was 6.9 kDa; it is conceivable that the six pare disulfides of CS5931 influenced the electrophoretic velocity and the SDS-PAGE molecular weight [11]. We then performed Western blotting analysis to further confirm the purified polypeptide. The results showed that a clear band appeared on the same molecular weight as the polypeptide from SDS-PAGE analysis (Figure 1b).

### 2.2. Optimization of Fermentation Conditions

**Figure 2:**
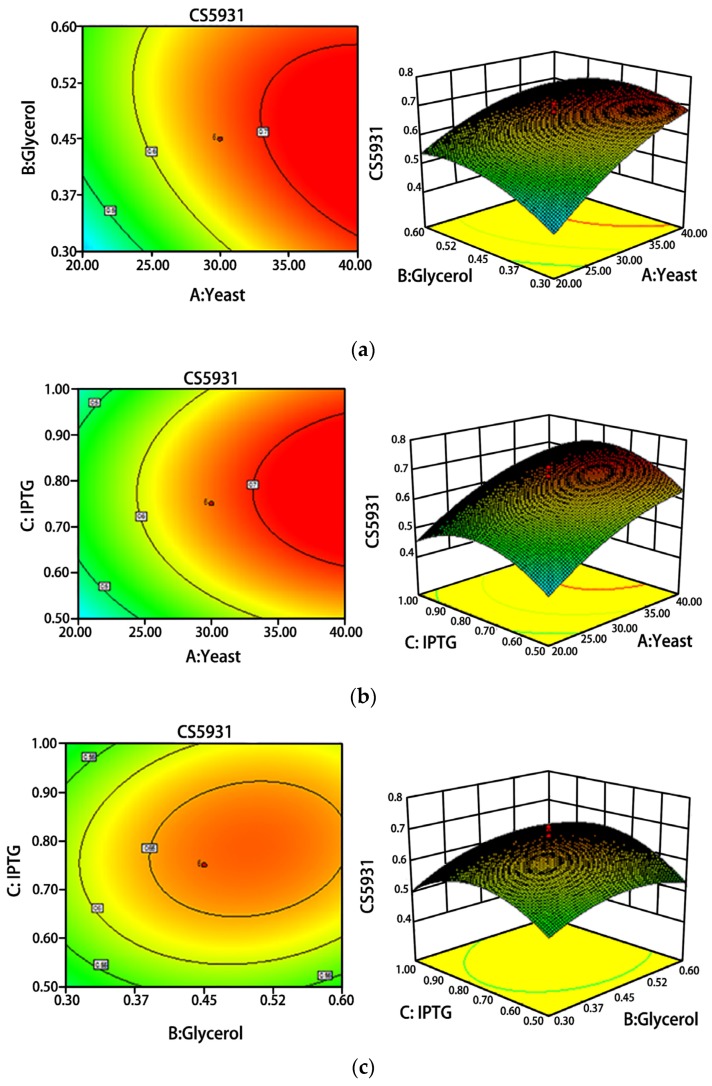
Response surface plots (3D) analysis showing the influence of three studied variables on the CS5931 production. (**a**) interaction between yeast extract concentration and glycerol concentration; (**b**) interaction between yeast extract concentration and IPTG concentration; (**c**) interaction between glycerol concentration and IPTG concentration.

Response surface approach was performed to study the effects of fermentation conditions on the production of CS5931, as shown in Figure 2. The best medium compositions were 10 g/L tryptone, 30 g/L yeast extract, 0.45% glycerol and 0.75 mM IPTG. We then used the optimized conditions for fermentation of CS5931, and the results revealed that the yield of CS5931 was increased by 73.33% compared with the ordinary LB medium (Figure 2). The yield of CS5931 was elevated from 2.0 to 7.5 mg/L after optimization.

### 2.3. Renaturation of CS5931

**Figure 3:**
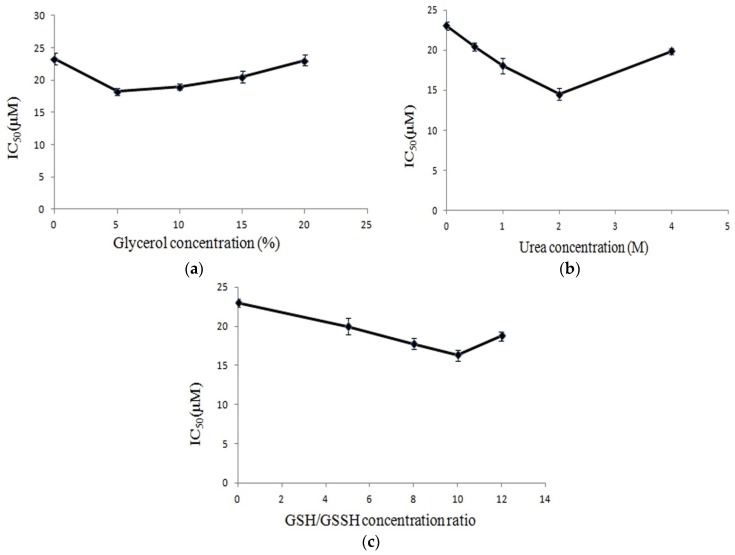
Effects of small molecules on the cytotoxicity of CS5931. Recombinant CS5931 was treated with certain renaturing conditions and the cytotoxicity of CS5931 on human colon carcinoma HCT116 cells was determined using MTT assay. (**a**) effect of glycerol on the cytotoxicity of CS5931; (**b**) effect of urea on the cytotoxicity of CS5931; (**c**) effect of GSH/GSSH on the cytotoxicity of CS5931.

Since the CS5931 consists of six pare disulfides, the polypeptide possesses a complicated 3D structure [13], and maintaining its intact structure is important for its antitumor activity. We next studied the effects of denaturing and renaturing conditions on the anticancer activity of CS5931. The cytotoxicity of recombinant CS5931 on human colon cancer HCT116 cells was analyzed using MTT assay. As shown in Figure 3a, addition of glycerol can improve the inhibitory activity of CS5931 on HCT116 cancer cells, and 5% glycerol is most remarkable, the IC_50_ decreased from 23.2 μM to 18.2 μM. The addition of urea could also improve the activity of CS5931 on HCT116 cancer cells; treatment with 2 M concentration of urea resulted in enhancement of the activity of the polypeptide significantly; the value of IC_50_ was decreased from 23.2 μM to 14.5 μM (Figure 3b). Similarly, as shown in Figure 3c, involvement of GSH/GSSH (2 mM/0.2 mM) in the dialysis buffer also ameliorated the activity of CS5931, the IC_50_ was decreased from 23.2 μM to 15.6 μM. We then used the optimized renaturing conditions to refold the polypeptide, and the IC_50_ of the polypeptide on human colon cancer HCT116 cells was decreased to 11.6 μM. These results revealed that optimization of renaturing conditions improves the activity of CS5931 significantly.

We next perform purification and renaturation using optimized fermentation and renaturation conditions. As shown in Table 1, the purity of CS5931 was increased to almost 10 times, and the activity of CS5931 was also enhanced significantly, using the optimized renaturation conditions (50 mM Tris-HCl, 0.5 M EDTA, 50 mM NaCl, 0.5 M l-arginine, 5% glycerol, 2 M urea, 2 mM/0.2 mM GSH/GSSH), the value of IC_50_ of CS5931 on HCT116 cancer cells was decreased to 11.6 μM from 23.2 μM. The study developed an effective approach for obtaining enough amount of CS5931.

### 2.4. CS5931 Inhibits the Growth of HCT116 Xenograft in Athymic Mice

**Figure 4:**
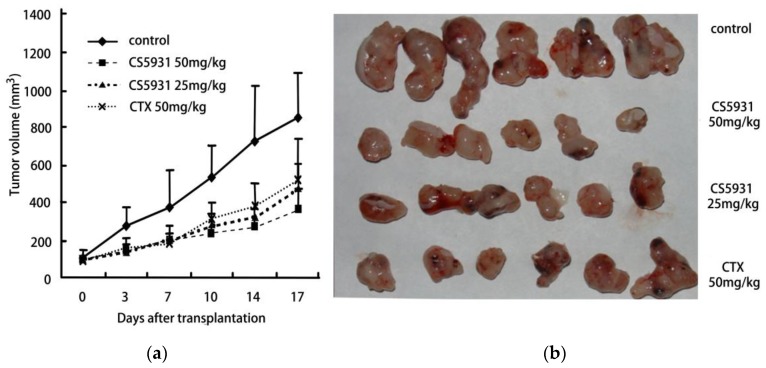
CS5931 inhibits the tumor growth in HCT116 xenograft athymic mice. The six to eight-week-old athymic nude mice (BALB/c, nu/nu) were divided into four groups, and treated with CS5931 by intraperitoneal injection every day for 10 days. (**a**) Tumor sizes were determined using a vernier caliper; (**b**) After being grown for another three weeks, all mice were euthanized, and tumors were removed and photographed.

In order to study the antitumor effect of CS5931 *in vivo*, experiments were performed with human colon cancer HCT116 xenograft in nude mice. As shown in Table 2 and Figure 4, CS5931 at 25 and 50 mg/kg dose levels displayed potent antitumor efficacies with 48% and 67% tumor growth inhibition (TGI) (*p* < 0.01, compared with control), respectively. The results suggested that CS5931 was able to inhibit the growth of tumor *in vivo* significantly in a dose-dependent manner.

## 3. Discussion

In this study, we make use of the response surface plots method to optimize the fermentation conditions for the production of CS5931; the production of the polypeptide is significantly increased. Furthermore, the denaturing and renaturation conditions were also studied. The activity of CS5931 was improved greatly using the optimized conditions. This study is important for developing CS5931 as a novel anticancer agent.

Response surface plots (3D) analysis is commonly used in optimizing the fermentation conditions of *E. coli*. The recombinant human interferon beta (rhIFN-β)-1b was optimized in shake flasks using response surface methodology (RSM). The production of rhIFN-β-1b was significantly increased using the optimized conditions, and the rhIFN-β production was increased from 0.255 g·L^−1^ to 0.981 g·L^−1^ [14]. In our present study, we use this approach to improve the fermentation conditions of CS5931, and the results show that the production of CS5931 is greatly increased; the yield of the purified polypeptide was increased from 2.0 mg/L to 7.5 mg/L. The results confirm that the response surface plots method is an effective approach for optimizing the fermentation conditions of *E. coli*.

CS5931 consists of 12 amino acid residues, and prediction of 3D structure of the polypeptide reveals that it forms two beta-hairpins, similar to human GRN-A [12]. Therefore, maintaining an intact three dimensional structure of CS5931 is necessary for its anticancer activity. Growing evidence shows that the choice of renaturing conditions plays an important role in preparing active renatured protein. Studies have shown that some small moleculars, like l-arginine [15], l-cysteine [16], EDTA, glycerol, urea, GSH/GSSH [17] are beneficial for protein refolding; these kinds of moleculars are able to inhibit the protein aggregation, abolish the degradation, improve the protein stability as well as interfere with the interaction of protein-protein. Redox agents, such as GSH/GSSH, are often used as accelerators for the correct formation of disulfide bonds [18,19,20]. In our present study, we found that the concentrations of glycerol, urea and GSH/GSSH affect the anticancer activity; using the optimized conditions, the value of IC_50_ of the polypeptide is decreased from 23.2 to 11.6 μM. Our study further reveals that selection of suitable concentrations of redox agents and glycerol is important for the right folding of CS5931. Our previous study has shown that CS5931 consists of parallel, stacked β-hairpins held together by six disulfide bonds. However, the relationship between the structure and activity as well as the formation of S-S bonds of the polypeptides need to be addressed.

His-tag is usually added at the N-terminal of protein for the easy purification. However, the tag sequences in most situations have to be removed for use clinically. In our present study, we construct an expression vector, called His-tag-DDDK-CS5931, which is able to express the fusion protein His-tag-DDDK-CS5931. Using the same optimized condition as that of the his-tag CS5931, the fusion protein was expressed in *E. coli*, and purified with Ni^2+^ column. Our results showed that the yield of His-tag-DDDK-CS5931 is similar to that of the his-tag CS5931. We then cut the fusion protein with enterokinase (New England Biolabs, Massachusetts, MA, USA), to obtain CS5931 without his-tag. Then, renaturation was performed with the optimized conditions. MTT assay reveals that the new polypeptide exhibits similar activity as the his-tag CS5931 (Supplementary materials, Figure S1).

In our previous study, we have found that CS5931 is able to induce cancer cell death via apoptotic pathways [12]. Here, we further confirm that the polypeptide inhibits the growth of tumors in nude mice. The results provide primary evidence that CS5931 possesses the potential to be developed as a novel anticancer agent. However, the targets of CS5931 have not been elucidated, and further studies to address the exact targets of the polypeptides are needed.

## 4. Materials and Methods

### 4.1. Recombinant Expression of CS5931 from E. coli BL21 (DE3)

The expression vector, pET28a/CS5931 synthesized by our lab previously [12], was transfected into *E. coli* BL21 (DE3) cells (Novagen, Darmstadt, Germany). Cells were incubated in low-salt LB medium (containing 100 µg/mL Kanamycin) at 37 °C with shaking at 200 rpm. After the value of OD_600_ of the culture medium reached around 0.5–0.7, IPTG was added at a final concentration of 0.75 mM and the cells were incubated overnight at 16 °C. Cells were collected by centrifugation (10,000 *g*, 10 min, 4 °C). The expression of recombinant protein was determined by SDS-PAGE (15% separating gel and 5% stacking gel).

### 4.2. Purification of the His-Tag CS5931 Polypeptide

The purification of CS5931 was performed using a 5 mL Ni^2+^-NTA column (GE Healthcare, Milwaukee, WI USA). In brief, *E. coli* BL21(DE3) cells (7 g, wet weight) were collected by centrifugation at 10,000 *g* for 10 min, and resuspended in cell lysis buffer (70 mL, containing 10 mM imidazole, 0.5 M NaCl, 20 mM Tris-HCl, pH8.0). The mixture was sonicated for 10 min on ice with interruption for 2 s every 4 s. The resulting cell lysate was centrifuged at 13,000 *g* for 30 min at 4 °C to remove the insoluble fraction. The supernatant was loaded onto a Ni^2+^ chelating sepharose column (GE Healthcare), equipped on AKTA. After washing the column with the washing buffer (60 mM imidazole, 0.5 M NaCl, 20 mM Tris-HCl, pH 8.0) for 3 times, the his-tag CS5931 was eluted with elution buffer (300 mM imidazole, 0.5 M NaCl, 20 mM Tris-HCl, pH8.0), The purified polypeptide was determined by 15% SDS-PAGE.

### 4.3. Optimization of Fermentation Conditions of CS5931 by Response Surface Methodology

The single factor experiments and Plackett-Bruman test were designed using the program Design-Expert, version 8.05b. Initial experiments found that three factors, including yeast extract, glycerol and IPTG concentration were the main factors influencing the expression of CS5931. After selection of the significant factors, central composite design (CCD) was used to evaluate the interaction of the independent value on CS5931 expression. A three-level three-factors CCD was selected to evaluate the interaction of these three variables, and the yeast extract concentration (factor A), glycerol concentration (factor B), and IPTG concentration (factor C) were coded as X_1_, X_2_, and X_3_, respectively. The minimum and maximum values of the yeast extract concentration were set at 20 and 40 g/L, while glycerol concentration is between 0.3% and 0.6%, and IPTG concentration is between 0.5 mM and 1.0 mM (Table 3). The complete design consisted of 20 combinations, including five replicates of the center point (Table 4). The experimental design and statistical analysis were all performed using the statistical package Design-Expert, version 8.05b [21,22,23,24,25].

### 4.4. Denaturing and Renaturing of the His-Tag CS5931 Polypeptide

The purified polypeptide, loaded in a dialysis bag (1 kDa), was denatured with urea at 8 M final concentration and refolded as the follows. The initial refolding buffer is composed of 50 mM Tris-HCl, 0.5 mM EDTA, 50 mM NaCl and 0.5 mM l-Arginine. A total of three kinds of dialysis conditions were set; condition 1 contained 0, 5, 10, 15, 20% concentrations of glycerol, while condition 2 contained 0, 0.5, 1.0, 2.0, 4.0 M concentrations of urea, and condition 3 contained 0, 5:1, 8:1, 10:1, 12:1 concentration ratios of GSH/GSSH. An MTT assay was carried out to determine the activity of CS5931 in each refolding condition and to evaluate the renaturing results.

### 4.5. Western Blotting Analysis

Western blotting analysis was performed as previously described [26]. Briefly, the purified CS5931 polypeptide was resolved on 15% SDS-PAGE. The gel was then semi-dry electroblotted onto a polyvinylidene fluoride membrane (Bio-Rad, Hercules, CA, USA) with 2.5 mA/cm^2^ for 20 min. The membrane then was then incubated in blocking buffer (5% fat milk in PBS) for 1 h at room temperature, washed with PBST for three times for 10 min each time, and incubated in PBST overnight at 4 °C with anti-his monoclonal antibody (anti-His 1:1000, Santa Cruz Biotechnology, Delaware, CA, USA). After washing the membrane with PBS, an HRP-conjugated antibody (goat anti-rabbit) was used as the secondary antibody and incubated for 1 h. The membrane was processed using the enhanced chemiluminescence method (Thermo, Waltham, USA), and the protein band was visualized by Gel imaging (Bio-Rad, Gel Doc XR^+^, Hercules, CA, USA).

### 4.6. Cell Proliferation Assay

MTT assay was performed to evaluate the anti-proliferative effects of the recombinant CS5931 against human colon carcinoma HCT116 cells. In order to eliminate the effect of solvents contained in CS5931, all of the polypeptides isolated from different batches were processed with the same conditions, dialyzed in PBS buffer (pH7.4, Hyclone, Logan, UT, USA) overnight and then ultrafiltrated with a 3 kDa ultrafiltration tube (Millipore, Billerica, MA, USA). Briefly, cells were plated on to 96-well-plates and incubated at 37 °C in humidified air atmosphere with 5% CO_2_. After incubation for 24 h, the cells were treated with or without certain concentrations of recombinant CS5931. After being cultured for another 48 h, MTT (20 μL, 5 mg/mL MTT in PBS, Sigma, San Francisco, CA, USA) was added to each well and the cells were incubated for an additional 4 h. DMSO (150 μL) was added to each well to dissolve the reduced MTT crystals. The MTT-formazan product dissolved in DMSO was estimated by measuring the absorbance at 570 nm with a microplate reader (Biotech, power wave, Los Angeles, AL, USA). All experiments were carried out in triplicate. The percentage of cell growth inhibition was calculated as follows:

Relative inhibition rate (%) = (OD_control group_ − OD_experimental group_)/OD_control group_ × 100%.
The relative viability rate (%) = 1 − relative inhibition rate × 100%.
The IC_50_ value was expressed as the concentration of drugs causing 50% inhibition.

### 4.7. Antitumor Activity in Vivo

Antitumor activity *in vivo* was carried out using HCT116 xenograft model. The 6 to 8-week-old athymic nude mice (BALB/c, nu/nu) were obtained from the Institute of Experimental Animals, Chinese Academy of Medical Sciences and Peking Union Medical College (Beijing, China). Exponentially growing cells were harvested, adjusted to 4 × 10^6^ per 200 μL and injected subcutaneously into the right axilla of each nude mouse. Seven days after postimplantation, the nude mice were randomly divided into four groups (six mice per group) as follows:

- Group A received normal saline;
- Group B received CTX 50 mg/kg as positive drug;
- Groups C and D received CS5931 with 25.0 and 50.0 mg/kg, respectively.

All mice were maintained in a pathogen-free isolator on hardwood bedding with a 12-h dark/light cycle, and with *ad libitum* access to the same standard chow. Each group was treated by intraperitoneal injection every day for 10 days.

Tumor sizes were recorded after postimplantation using a vernier caliper. After being grown for another 3 weeks, all mice were euthanized. Then, the final body and tumor weight were recorded and the mean weights were calculated. The TGI for each group was calculated using the following formula: TGI = (1 − mean treated tumor weight/mean control tumor weight) × 100%. The tumor tissues were snap-frozen in liquid nitrogen and stored at −80 °C for Western blot analysis. The animal experiments were approved by the Experimental Animal Center of Shandong province, China, and the mice were treated in accordance with international animal ethics guidelines.

## 5. Conclusions

In the present study, CS5931 was expressed and purified the polypeptide from *E. coli*, and the fermentation conditions were studied using response surface methodology. The yield of CS5931 was increased significantly. The denaturing and renaturation conditions were also studied. Using the optimized renaturation condition. The activity of CS5931 was improved greatly using the optimized conditions. *In vivo* study using xenograft nude mice bearing HCT116 cancer cells revealed that CS5931 was able to inhibit the growth of tumor significantly. This study is provide primary evidence that CS5931 have potential to be developed as a novel anticancer agent.

## Figures and Tables

**Table 1:** Purification of recombinant CS5931 from *E. coli* BL 21 with optimized conditions.

Purification Steps	Total Protein (mg)	CS5931 (mg)	Yield (%)	Purity (%)	IC_50_ (μM)
Cell lysate	539	54	-	10	-
Ni-NTA column	48.5	47	9	97	23.2
Protein renaturation	24.3	23.8	50	98	-
Protein frozen-dried	7.65	7.5	32	98	11.6

One liter culture of *E. coli* BL21 (DE3) was used in the purification with the optimized medium and renaturation conditions. The content of polypeptide was measured by the method of BCA (Kaiji, Beijing, China, BCA protein assay kit). Total protein was resolved by SDS-PAGE and the content of CS5931 was analyzed by BandScan software. The value of IC_50_ of CS5931 was analyzed using MTT assay.

**Table 2:** Growth inhibition of CS5931 in HCT116 xenograft in athymic mice.

Group	Dosage (mg/kg)	No. of Mice (Begin/End)	Body Weight Change (g)	Tumor Weight (mean ± SD, g)	%TGI
Control	-	6/6	+4.2	1.35 ± 0.20	-
CTX	50	6/6	+0.9	0.63 ± 0.13	54 **
CS5931	25	6/6	+1.2	0.69 ± 0.13	48 **
CS5931	50	6/6	+0.4	0.45 ± 0.14	67 **

Data are represented as the mean ± SD of three independent experiments, cyclophosphamide (CTX), tumor growth inhibition (TGI), ** *p* < 0.01 compared with control. No mouse died during the experimental period.

**Table 3:** Independent variables and their coded and actual values used for optimization.

Independent Variables	Symbol	Code Levels	
		−1	1
Yeast extract concentration, g·L^−1^	X_1_	20	40
Glycerol concentration, %	X_2_	0.3	0.6
IPTG concentration, mM	X_3_	0.5	1.0

The three factors including the yeast extract concentration (factor A), glycerol concentration (factor B), and IPTG concentration (factor C) were set in different values.

**Table 4:** Central composite design of the fermentation conditions.

Experiment	Factor A, g·L^−1^	Factor B, %	Factor C, mM	CS5931, mg
	X_1_	X_2_	X_3_	
1	40.00 (1)	0.30 (−1)	1.00 (1)	0.708
2	20.00 (−1)	0.60 (1)	1.00 (1)	0.474
3	20.00 (−1)	0.30 (−1)	1.00 (1)	0.370
4	40.00 (1)	0.60 (1)	0.50 (−1)	0.538
5	30.00 (0)	0.45 (0)	0.75 (0)	0.680
6	30.00 (0)	0.45 (0)	0.75 (0)	0.659
7	46.82 (1.682)	0.45 (0)	0.75 (0)	0.700
8	30.00 (0)	0.45 (0)	0.75 (0)	0.681
9	40.00 (1)	0.60 (1)	1.00 (1)	0.610
10	30.00 (0)	0.45 (0)	0.33 (−1.682)	0.423
11	30.00 (0)	0.45 (0)	1.17 (1.682)	0.401
12	30.00 (0)	0.45 (0)	0.75 (0)	0.597
13	40.00 (1)	0.30 (−1)	0.50 (−1)	0.627
14	20.00 (−1)	0.30 (−1)	0.50 (−1)	0.378
15	30.00 (0)	0.45 (0)	0.75 (0)	0.700
16	30.00 (0)	0.45 (0)	0.75 (0)	0.709
17	30.00 (0)	0.20 (−1.682)	0.75 (0)	0.348
18	20.00 (−1)	0.60 (1)	0.50 (−1)	0.340
19	13.18 (−1.682)	0.45 (0)	0.75 (0)	0.392
20	30.00 (0)	0.70 (1.682)	0.75 (0)	0.671

Central composite design of the fermentation conditions was designed using Design-Expert, version 8.05b, and the paired number indicated the different combination of fermentation conditions.

## Supplementary Files

Supplementary File 1
