# Supplementary material for: Preparation and Antitumor Activity of CS5931, A Novel Polypeptide from Sea Squirt Ciona Savignyi
Source: Mar Drugs. 2016 Mar 21;14(3):47. doi: 10.3390/md14030047 (PMC4820301; doi:10.3390/md14030047)
Supplement: Supplementary File 1 [file marinedrugs-14-00047-s001.pdf]

Supplementary Materials: Preparation and Antitumor Activity of CS5931, a Novel Polypeptide from Sea Squirt *Ciona Savignyi*

Xiaoshuang Chen, Huanli Xu, Bo Li, Feng Wang, Xiaoliang Chen, Dexin Kong and Xiukun Lin

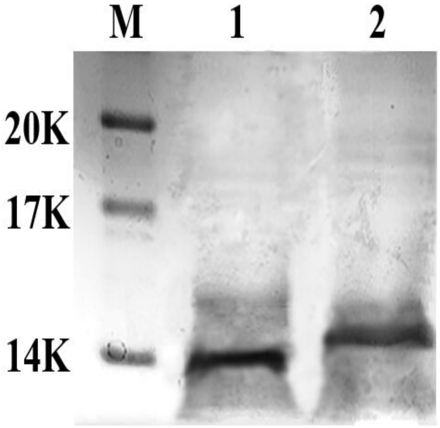

(**a**)

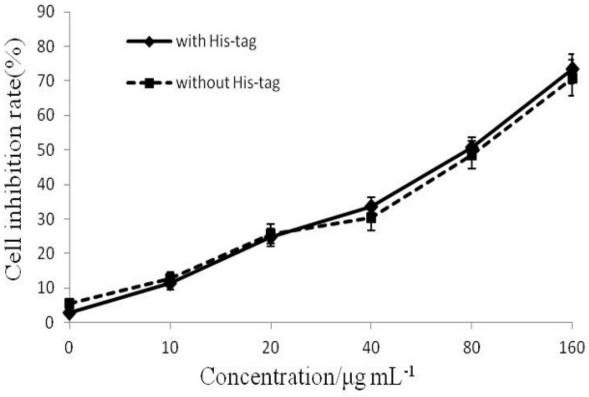

(**b**)

Figure S1. Purification and cytotoxicity of fusion protein His-tag-DDDK-CS5931. The expression plasmid, pET28a/His-tag-DDDK-CS5931 was constructed, and transformed into *E. Coli* BL21 cells. The strain was cultured using optimized conditions. After purification using Ni^{2+} column, the polypeptide was cut using enterokinase, and renatured using the optimized renaturation conditions. (**a**) SDS-PAGE analysis. Lane 1 represented inhibitory effect of CS5931 without his-tag on HCT116 cancer cells while lane 2 indicated the effect of his-tag CS5931; (**b**) Comparison of the cytotoxicity of his-tag CS5931 with CS5931 without his-tag on human colon carcinoma HCT116 cells. MTT assay was performed to determine the cytotoxicity of the polypeptides.
